# Comparative Transcriptomic Analysis of Immune-Related Gene Expression in Duck Embryo Fibroblasts Following Duck Tembusu Virus Infection

**DOI:** 10.3390/ijms19082328

**Published:** 2018-08-08

**Authors:** Guanliu Yu, Yun Lin, Yi Tang, Youxiang Diao

**Affiliations:** 1 College of Animal Science and Technology, Shandong Agricultural University, 61 Daizong Road, Tai’an 271018, Shandong, China; yuguanliu@163.com (G.Y.); lyun1994@126.com (Y.L.); 2 Shandong Provincial Key Laboratory of Animal Biotechnology and Disease Control and Prevention, Shandong Agricultural University, 61 Daizong Road, Tai’an 271018, Shandong, China; 3 Shandong Provincial Engineering Technology Research Center of Animal Disease Control and Prevention, Shandong Agricultural University, 61 Daizong Road, Tai’an 271018, Shandong, China

**Keywords:** RNA-seq, DTMUV, virus infection, duck embryo fibroblast, transcriptome

## Abstract

Duck is a major waterfowl species in China, providing high-economic benefit with a population of up to 20–30 billion per year. Ducks are commonly affected by severe diseases, including egg-drop syndrome caused by duck Tembusu virus (DTMUV). The immune mechanisms against DTMUV invasion and infection remain poorly understood. In this study, duck embryo fibroblasts (DEFs) were infected with DTMUV and harvested at 12 and 24 h post-infection (hpi), and their genomes were sequenced. In total, 911 (764 upregulated and 147 downregulated genes) and 3008 (1791 upregulated and 1217 downregulated) differentially expressed genes (DEGs) were identified at 12 and 24 hpi, respectively. Kyoto Encyclopedia of Genes and Genomes enrichment analysis revealed that DEGs were considerably enriched in immune-relevant pathways, including Toll-like receptor signaling pathway, Cytosolic DNA-sensing pathway, RIG-I-like receptor signaling pathway, Chemokine signaling pathway, NOD-like receptor signaling pathway, and Hematopoietic cell lineage at both time points. The key DEGs in immune system included those of the cytokines (*IFN α2*, *IL-6*, *IL-8L*, *IL-12B*, *CCR7*, *CCL19*, and *CCL20*), transcription factors or signaling molecules (*IRF7*, *NF-κB*, *STAT1*, *TMEM173*, and *TNFAIP3*), pattern recognition receptors (*RIG-I* and *MDA5*), and antigen-presenting proteins (*CD44* and *CD70*). This suggests DTMUV infection induces strong proinflammatory/antiviral effects with enormous production of cytokines. However, these cytokines could not protect DEFs against viral attack. Our data revealed valuable transcriptional information regarding DTMUV-infected DEFs, thereby broadening our understanding of the immune response against DTMUV infection; this information might contribute in developing strategies for controlling the prevalence of DTMUV infection.

## 1. Introduction

Hemorrhagic ovarian inflammation, also termed egg-drop syndrome, is primarily caused by duck Tembusu virus (DTMUV), and has resulted in significant economic losses in the poultry industry in Southeast Asia since 2010 [1]. DTMUV, similar to other flaviviruses, is a single-stranded, positive-sense RNA virus with an approximately 11 kb genome. The virus belongs to the Ntaya antigenic group of the family Flavirviridae [2]. It infects ducks in addition to geese [3,4], chickens [5], sparrows [6], mice [7,8], and pigeons [9]. More importantly, a wide spectrum of mammalian cells exhibit cytopathic effects (CPEs) following Tembusu virus infection, such as A549, BHK21, Hela, Vero, and SH-SY5Y [10], and also including HEK293 according to our recent study. These findings imply that the virus has expanded its host range and may pose a threat to mammals’ health [11].

Better understanding of the immune responses to DTMUV invasion and infection in host cells is essential for developing strategies to control infection by the virus. Once the virus invades host cells, the first line of cellular defense (i.e., host innate immune system) involves the activation of pathogen-associated molecular patterns by pattern recognition receptors (PRRs), including toll-like receptors, nucleotide oligomerization domain (NOD)-like receptors, and retinoic acid-inducible gene I (RIG-I)-like receptors. Subsequently, interferon (IFN)-related signaling pathways are activated, resulting in the induction of IFN-stimulated genes, which can inhibit viral replication [12]. Therefore, a broadened understanding of the host immune system will be beneficial for better comprehension the molecular mechanisms underlying pathogen-host interaction.

Notably, high-throughput RNA-sequencing (RNA-Seq) technology, which has the advantages of broad genome coverage, speed, low-cost, high precision, and unbiased quantification of transcript expression, has been widely used to explore the molecular mechanisms underlying pathogen-host interactions in recent years [13,14,15,16,17,18,19]. Transcriptome analysis via RNA-Seq can reveal all RNA transcripts in tissues or cells and further elucidate the expression of genes in specific tissues or cells in different physiological states and cellular environment [20].

To date, DTMUV-related studies have focused on pathogenicity [21,22], detection methods [23,24], vaccines [10,25,26], transmission routes [27], genetic evolution and epidemiology [1,28,29], and molecular structure [30,31]. Unfortunately, the immune mechanisms of DTMUV invasion and infection in host cells remain poorly understood. Therefore, to acquire transcriptome information and to study the interactions of DTMUV with host cells, duck embryo fibroblasts (DEFs) were infected with DTMUV, harvested at 12 and 24 h post-infection (hpi), and subjected to transcriptome sequencing. Gene Ontology (GO) and Kyoto Encyclopedia of Genes and Genomes (KEGG) enrichment analyses were used to examine differentially expressed genes (DEGs) identified during different stages of infection. Our findings expand our understanding of the immune mechanisms underlying DTMUV infection.

## 2. Results

### 2.1. DTMUV Infection Induces Cytopathic Effects (CPEs) and Reduces Cell Viability

**Figure 1:**
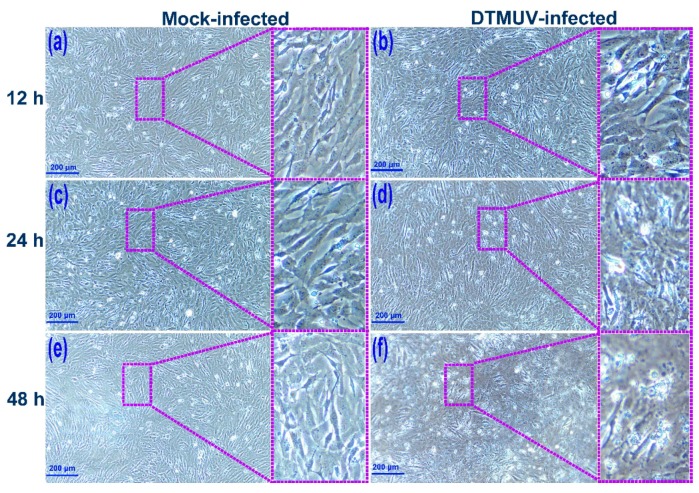
Cytopathic effects following infection of duck embryo fibroblasts with duck Tembusu virus at 12, 24, and 48 h post-infection. (**a**,**c**,**e**) no CPEs were observed in mock-infected cells. (**b**,**d**,**f**) the CPEs (e.g., cell shrinkage, rounding, and suspension) were found in cells infected with DTMUV. The blue scale bar represents 200 μm.

As shown in Figure 1, no CPEs were observed in mock-infected cells (Figure 1a,c,e). However, in cells infected with DTMUV, CPEs (e.g., cell shrinkage, rounding, and suspension) were observed as early as 24 hpi (Figure 1d). At 48 hpi, most cells detached from culture vessel (Figure 1f).

**Figure 2:**
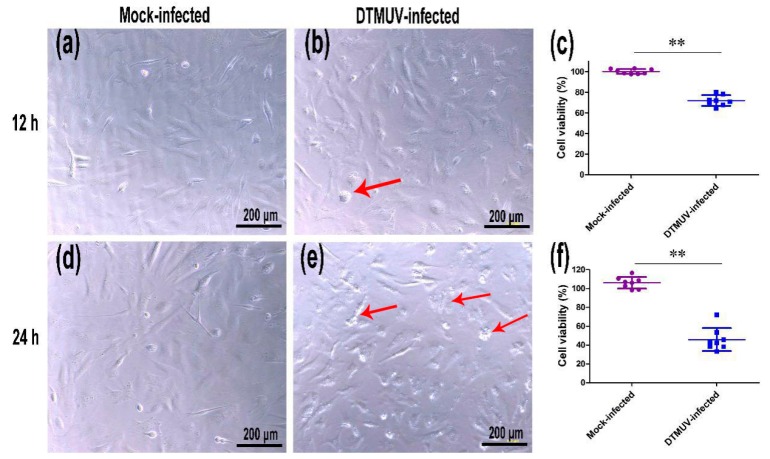
Viability of duck embryo fibroblasts at 12 (**a**–**c**) and 24 h (**d**–**f**) after infection with duck Tembusu virus (DTMUV). CPEs (cell shrinkage and rounding, red arrow) were observed in Figure 2b,e. Cell viability is shown as the mean ± SD of eight replicates.** *p* < 0.01.

The WST-1 cell proliferation assay was performed to examine cell viability following viral infection. As shown in Figure 2, mock-infected DEFs displayed nearly 100% viability at 12 and 24 hpi. Conversely, the viabilities of DTMUV-infected DEFs were 72.05% and 45.82% at 12 and 24 hpi (Figure 2c,f), respectively, confirming the high efficiency of viral infection.

### 2.2. Transcriptome Sequencing Data

As shown in Table 1, the raw reads, clean reads, clean bases, Q30, and total mapped percentage of each sample were recorded for each library. For all libraries, the percentages of clean data, Q20, and Q30 were >98%, >97%, and >94%, respectively. These results illustrated the high quality of the sequencing data and ensured their suitability for the next step of the analysis. All sequencing data have been uploaded to the Sequence Read Archive of the National Center for Biotechnology Information (accession number SRP150572).

**Figure 3:**
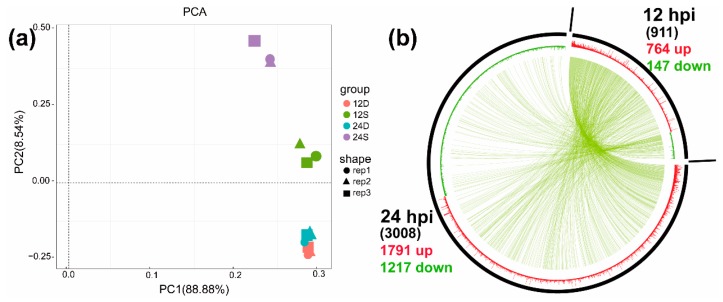
Principal component of samples (**a**) and visibility graph of differentially expressed genes (**b**) at 12 and 24 h post-infection (hpi). Note: (**a**): PC1 shows the differences among duck Tembusu virus (DTMUV)-infected samples; PC2 indicates differences between mock- and DTMUV-infected samples. 12D, mock-infected DEFs at 12 hpi; 24D, mock-infected DEFs at 24 hpi; 12S, DTMUV-infected DEFs at 12 hpi; 24S, DTMUV-infected DEFs at 24 hpi. (**b**): Upregulated differentially expressed genes (DEGs) are shown in red, whereas downregulated DEGs are shown in green. The green curve in the circle represents the common DEGs at 12 and 24 hpi.

In addition, principal component analysis was conducted on the basis of the expression level of each sample. As shown in Figure 3a, mock-infected cells (i.e., 12D and 24D) were clearly distinct from those infected with DTMUV (i.e., 12S and 24S). For DTMUV-infected samples, the correlation coefficients were proportional to the time post-infection; in other words, samples at 12 hpi did not cluster with those at 24 hpi. Hence, the infection efficiency of samples was different at 12 and 24 hpi.

### 2.3. Identification and Analysis of DEGs

In this study, after DEFs were infected with DTMUV, DEGs between the mock- and DTMUV-infected groups were analyzed under the threshold of *p*-value < 0.05 & |log2(fold change)| > 1. As shown in Figure 3b, 911 (764 upregulated and 147 downregulated genes) and 3008 (1791 upregulated and 1217 downregulated genes) DEGs were identified at 12 and 24 hpi, respectively. The details of the DEGs are listed in Table S1.

### 2.4. GO and KEGG Enrichment Analysis

**Figure 4:**
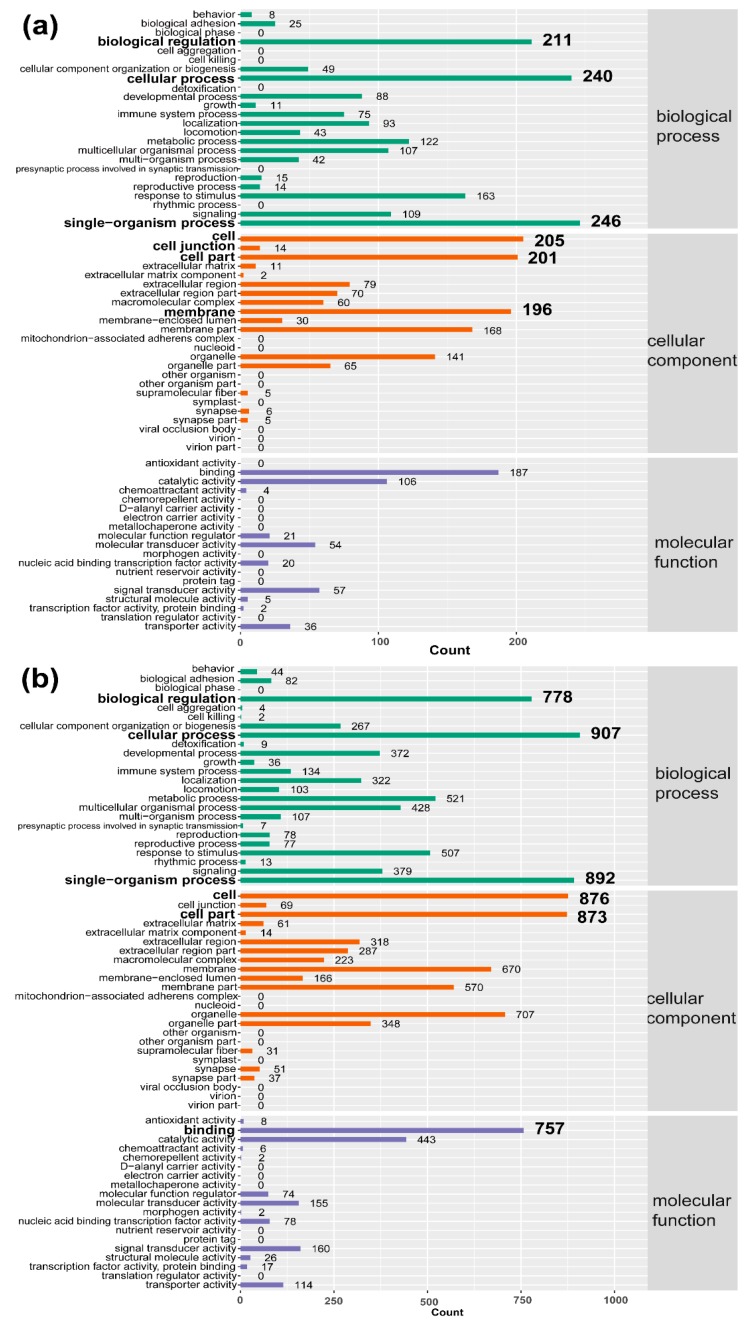
Gene Ontology enrichment of differentially expressed genes (**a**): 12 h post-infection (hpi); (**b**): 24 h post-infection 24 hpi.

GO is a standardized system for categorizing genes, and it is often used to explore the roles of DEGs. As shown in Figure 4, DTMUV infection was mainly related to single-organism processes, cellular processes, biological regulation, cellular components, and membrane binding at 12 and 24 hpi. Besides, the details of the GO terms are included in Table S2.

**Figure 5:**
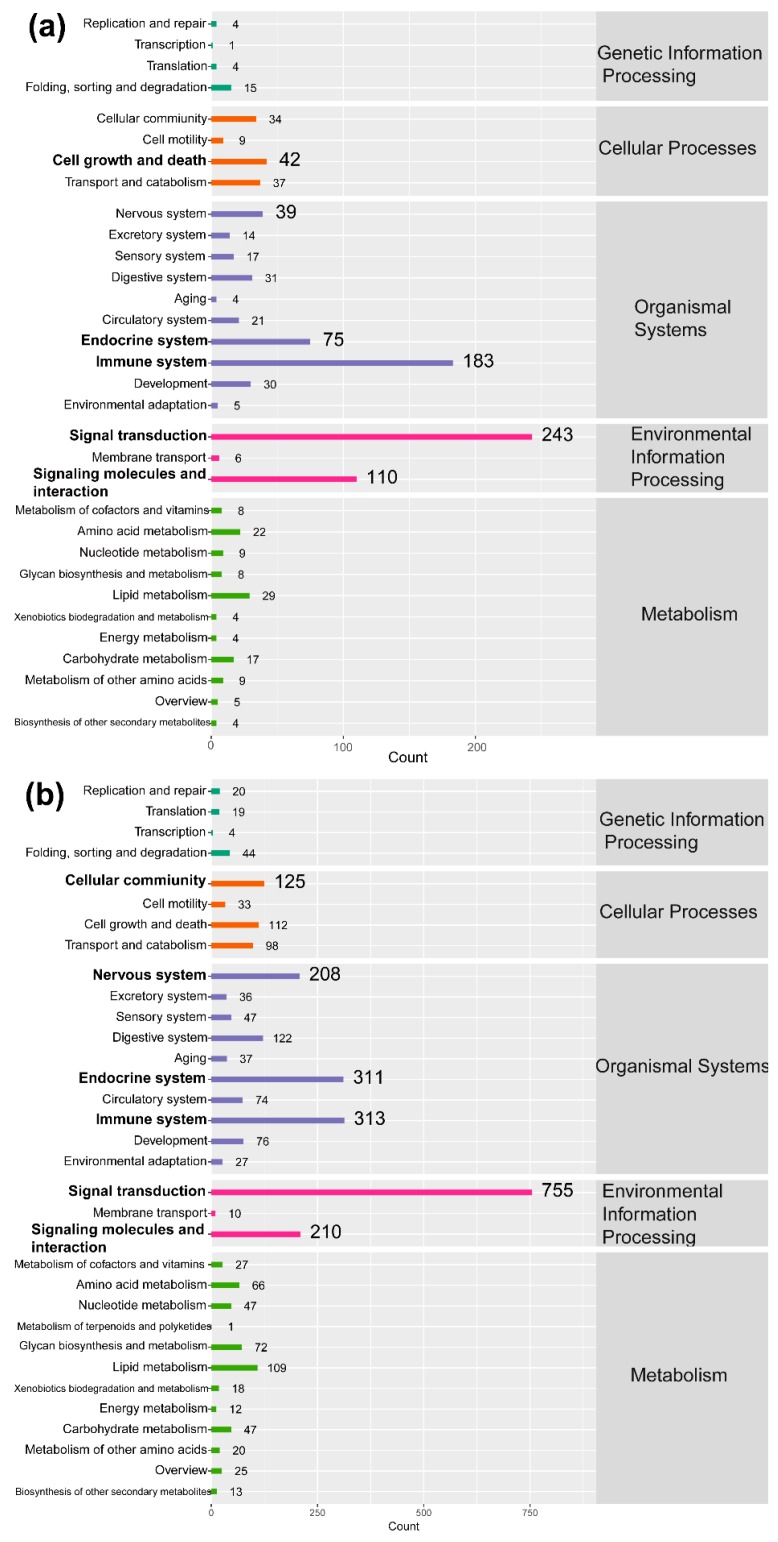
Kyoto Encyclopedia of Genes and Genomes (KEGG) enrichment analysis at 12 (**a**) and 24 h post-infection (**b**). The top six enriched KEGG terms at both the time points are marked in black bold font.

Furthermore, to further explore the functions of DEGs post DTMUV infection in DEFs, KEGG enrichment analysis was also conducted. As shown in Figure 5, DEGs were mainly enriched in the categories of signal transduction, immune system, signaling molecules and interaction, endocrine system, nervous system, and cellular community/cell growth and death at 12 and 24 hpi. The number of enriched immune system pathways was higher than those for other pathways, excluding signal transduction. Moreover, the details of the KEGG terms are included in Table S3.

### 2.5. Signaling Pathways in the Immune System

**Figure 6:**
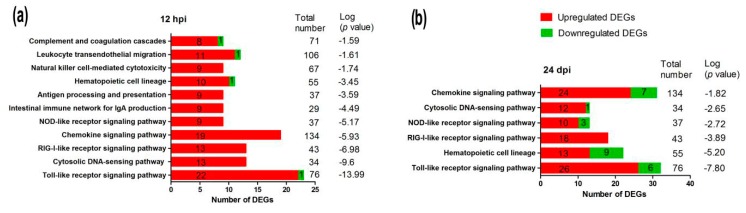
The immune system pathways enriched during duck Tembusu virus infections at 12 h (**a**) and 24 h (**b**) post-infection (hpi) (*p* < 0.05).

In this study, the DEGs were considerably enriched in immune-relevant pathways, including the Toll-like receptor signaling pathway, Cytosolic DNA-sensing pathway, RIG-I-like receptor signaling pathway, Chemokine signaling pathway, NOD-like receptor signaling pathway, and Hematopoietic cell lineage, at 12 and 24 hpi (Figure 6). As shown in Figure 6, 95.7% (22/23) of DEGs were upregulated in Toll-like receptor pathways at 12 hpi compared with 81.3% (26/32) of DEGs at 24 hpi. In Cytosolic DNA-sensing pathways, 100% (13/13) of DEGs were upregulated at 12 hpi compared with 92.3% (12/13) at 24 hpi. In Chemokine signaling pathways, 100% (19/19) of DEGs were upregulated at 12 hpi compared with 77.4% (24/31) at 24 hpi. In NOD-like receptor signaling pathways, 100% (9/9) of DEGs were upregulated at 12 hpi compared with 76.9% (10/13) at 24 hpi. Regarding DEGs involved in the Hematopoietic cell lineage, a higher percentage of DEGs were upregulated at 12 hpi than at 24 hpi (90.9% (10/11) vs. 59.9% (13/22)). Cumulatively, these results indicated that stronger immune responses were induced by DTMUV infection at 12 hpi than at 24 hpi. Detailed information about the major immune-relevant enriched genes is shown in Table S4.

### 2.6. Expression of Key DEGs in Immune-Related Pathways

**Figure 7:**
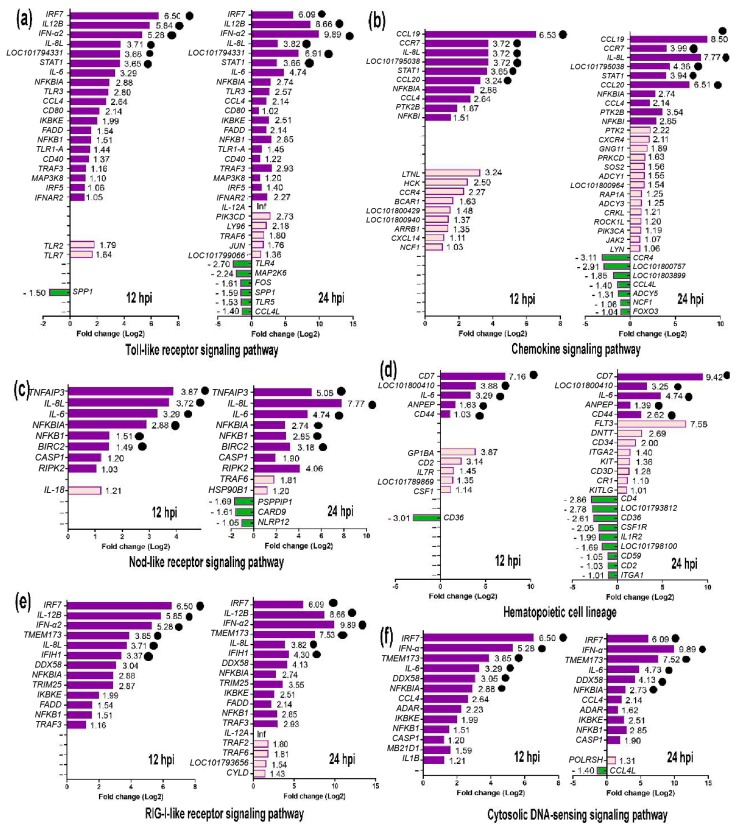
Differentially expressed genes (DEGs) in six common immune system-related signaling pathways at 12 and 24 h post-infection (hpi). (**a**) DEGs in Toll-like receptor signaling pathway at 12 and 24 hpi. (**b**) DEGs in Chemokine signaling pathway at 12 and 24 hpi. (**c**) DEGs in Nod-like receptor signaling pathway at 12 and 24 hpi. (**d**) DEGs in Hematopoietic cell lineage at 12 and 24 hpi. (**e**) DEGs in RIG-I-like receptor signaling pathway at 12 and 24 hpi. (**f**) DEGs in Cytosolic DNA-sensing signaling pathway at 12 and 24 hpi. The six DEGs with the largest fold changes in expression in each pathway are marked by black dots.

**Figure 8:**
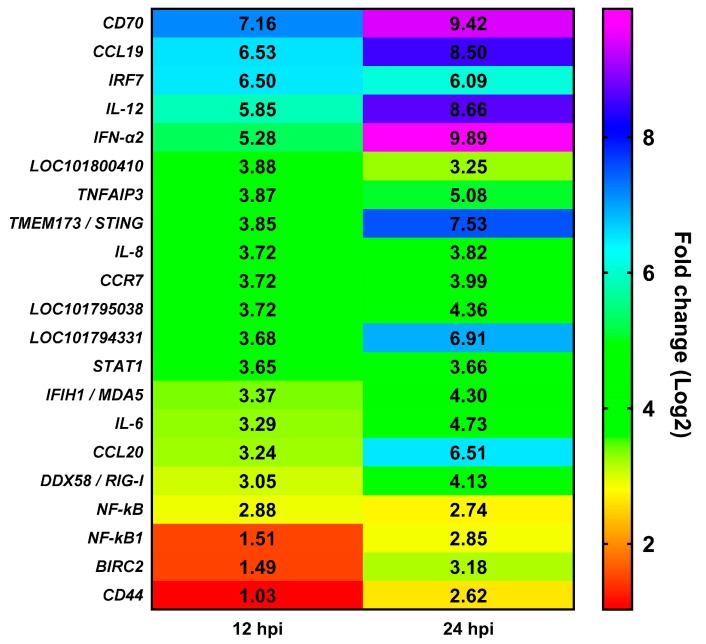
The top six differentially expressed genes in the Toll-like receptor signaling pathway, Chemokine signaling pathway, Nod-like receptor signaling pathway, Hematopoietic cell lineage, RIG-I-like receptor signaling pathway, and Cytosolic DNA-sensing signaling pathway. The colors in the grid represent the expression fold change as indicated by the color scale. Note: *LOC101794331* (T-lymphocyte activation antigen CD86-like); *LOC101795038* (fractalkine-like); *LOC101800410* (T-cell surface glycoprotein CD1b-3-like).

Genes in immune-related pathways with large fold changes in expression may play important roles in the process of host resistance to virus invasion. As shown in Figure 7, the top six DEGs in the Toll-like receptor signaling pathway were IFN stimulating factor 7 (*IRF7*), interleukin (*IL)*-*12B*, *IFN-α2*, *IL-8L*, *LOC101794331* (T-lymphocyte activation antigen CD86-like), and signal transducer and activator of transcription 1 (*STAT1*). For the chemokine signaling pathway, the top six DEGs were *CCL19, CCR7*, *IL-8L*, *LOC101795038* (fractalkine-like), *STAT1*, and *CCL20*. For the NOD-like receptor signaling pathway, the top six DEGs were *TNFAIP3*, *IL-8L*, *IL-6*, *NFKBIA*, *NFKB1*, and *BIRC2*. For the hematopoietic cell lineage, the top five DEGs were *CD7*, *LOC101800410* (T-cell surface glycoprotein CD1b-3-like), *IL-6*, *ANPEP*, and *CD44*. For the RIG-I-like receptor signaling pathway, the top six DEGs were *IRF7*, *IL-12B*, *IFN-α2*, transmembrane protein 173 (*TMEM173/STING*), *IL-8L*, and *IFIH1*. For the Cytosolic DNA-sensing signaling pathway, the top six DEGs were *IRF7*, *IFN-α2*, *TMEM173*, *IL-6*, *DDX58*, and *NFKBIA*. After ranking DEGs by expression at 12 hpi, it was apparent that immune-related DEGs mainly included cytokines (*IFN-α2*, *IL-6*, *IL-8L*, *IL-12B*, *CCR7*, *CCL19*, and *CCL20*), transcription factors or signaling molecules (*IRF7*, *nuclear factor κB*, *STAT1*, *TMEM173/STING*, and *TNFAIP3*), PRRs (*RIG-I*/*DDX58* and *MDA5*/*IFIH1*), and antigen-presenting proteins (*CD44* and *CD70*) (Figure 8). Details of these DEGs at 12 and 24 hpi are summarized in Table S4.

### 2.7. Validation of the Transcriptomic Data by Quantitative Real-Time (qRT)-PCR

**Figure 9:**
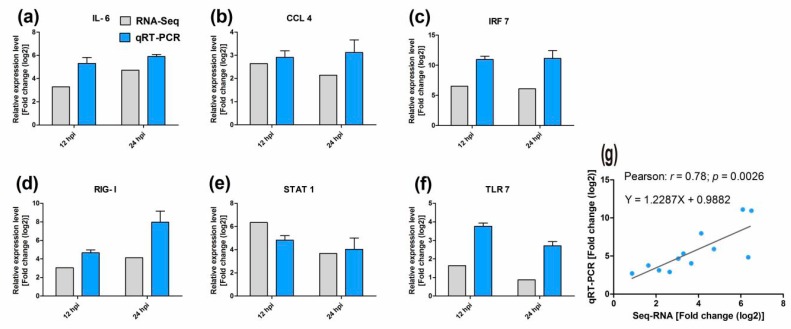
Confirmation of the transcriptome sequencing data by quantitative real-time (qRT)-PCR. Six differentially expressed genes (DEGs) involved in immune-related pathways were selected for qRT-PCR, and expression was estimated using the 2^−∆∆CT^ method. The qRT-PCR data are presented as the mean ± standard deviation (*n* = 5) (**a**–**f**). Pearson’s correlation analyses of the expression of above six DEGs between Seq-RNA and qRT-PCR (**g**).

To further verify the expression profiles of DEGs generated by RNA-Seq, six DEGs in the Toll-like receptor pathway were selected randomly for qRT-PCR analysis. The expression levels of the DEGs are shown in Figure 9.

Overall, the expression patterns of all six DEGs obtained via qRT-PCR were consistent with those obtained via RNA-Seq; however, the relative expression levels were not completely identical (e.g., *CCL4* in Figure 8b,). Furthermore, the correlation of the expression of the six DEGs between RNA-Seq and qRT-PCR was also analyzed using Pearson’s correlation analysis. The analysis revealed a Pearson’s correlation coefficient of 0.78 (*p* = 0.0026), confirming that the RNA-Seq data were reliable and accurate (Figure 9g).

### 2.8. Determination of Cytokine Levels in Culture Supernatant

**Figure 10:**
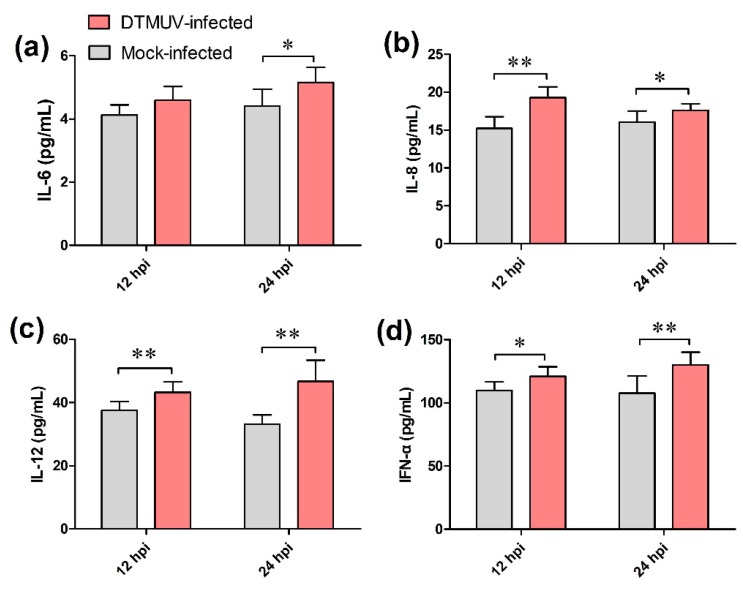
Concentrations of IL-6 (**a**); IL-8 (**b**); IL-12 (**c**); and IFN-α (**d**) in the culture supernatant of the mock- and duck Tembusu virus (DTMUV)-infected groups. The data are presented as the mean ± standard deviation (*n* = 6). * *p* < 0.05, ** *p* < 0.01.

Compared with the mock-infected group, the expression of IL-6, IL-8, IL-12, and IFN-α was significantly upregulated in the culture supernatant of the DTMUV-infected group at 12 and 24 hpi (*p* < 0.05 or *p* < 0.01, Figure 10). These data further confirmed the high expression of cytokines induced by DTMUV and reflected the reliability of the data generated by RNA-Seq.

## 3. Discussion

DTMUV is an important pathogen that endangers waterfowl, and it has caused loss of several billion dollars in the Chinese poultry industry since 2010. Therefore, it is of great significance to deepen our understanding of the molecular mechanisms underlying pathogen-host interactions to control the occurrence and prevalence of DTMUV infection. Over the past decade, RNA-Seq technology has emerged as a revolutionary and powerful tool for revealing the molecular expression profile and specific biological processes of disease development [32]. A well-known of molecular expression profile will contribute to clarity the molecular mechanisms between pathogen and host. However, to the best of our knowledge, no report has examined the molecular expression profile of the interaction between DTMUV and DEFs, excluding a transcriptome study of the spleens of DTMUV-infected goslings [4].

In this study, to better reflect the process by which DTMUV invades host cells, DEFs were selected as an in vitro model to investigate the molecular expression profile at the cellular level. It is noteworthy that the virus usually attacks immune components in host cells during the early or middle stages of infection, and a broadened understanding of the host immune system will help us better understand the molecular mechanisms underlying pathogen–host interaction [12]. For this reason, we primarily analyzed the effect of viral invasion on the host immune system at 12 and 24 hpi.

The innate immune response is crucial for fighting viral invasion during early-stage infection. Here, several cellular signaling pathways were identified to be involved in the response to DTMUV infection at 12 and 24 hpi, including Toll-like receptor signaling pathway, Cytosolic DNA-sensing pathway, RIG-I-like receptor signaling pathway, Chemokine signaling pathway, NOD-like receptor signaling pathway, and Hematopoietic cell lineage, and so on (Figure 6). Of the above signaling pathways, pattern recognition receptors (PRRs), including toll-like receptors (TLRs), RIG-I-like receptors, and NOD-like receptors, play a central role in recognizing viral RNA and initiating the innate immune response by activating type I IFN and inflammatory cytokines [4,33].

In this study, some key PRRs in the above mentioned pathways were recognized by DTMUV, such as RIG-I, and MDA5, which triggered the host innate immune response. As previously reported, MDA5 and RIG-I can recognize long RNA fragments or dsRNAs (not triphosphorylated) and 5′-triphosphorylated or short RNA fragments, respectively [5,34]. The two PRRs were also recognized by other flaviviruses, such as Dengue virus [35,36], Zika virus [34,37], Hepatitis virus [38], Japanese encephalitis virus [35,39], West nile virus [40,41], and Avian tembusu virus [5], indicating that DTMUV may trigger the same innate immune signaling pathway as the above flaviviruses. In this study, DTMUV was sensed by PRRs in DEFs, and various immune molecules were efficiently induced. The results were somewhat similar to those obtained in a previous study, which uncovered that *IFN-α*, *IFN-β*, *IFN-γ*, *IL-1β*, *IL-2*, *IL-6*, and *IL-8* were upregulated in the spleens of DTMUV-infected ducks [33]. However, these cytokines could not inhibit DTMUV replication in DEFs. This may be the reason for the occurrence of “cytokine storm”, which is a phenomenon of multiple inflammatory cytokines (e.g., TNF-α, IL-2, IL-8, IL-12, IFN-α, IFN-β, IFN-γ, and MCP-1, etc.) overproduced for resisting pathogens. The overexpressed cytokines can also cause great harm to the host when they resist pathogen invasion [33,42].

It is well-known that the transcription of innate immune molecules is depends on the activation of several transcriptional factors [5,43], such as IRF, NF-κB, and STAT. IRF7, is activated by TLR-dependent signaling pathways and it regulates type I IFN responses [44]. NF-κB is a vital transcription factor that regulates genes responsible for a variety of immune response [42]. STAT1, is one of the most important members of the STAT protein family, which plays important roles in regulating cell growth, proliferation, differentiation, and apoptosis [45]. In addition, inactivation of NF-κB or disruption of IRF7 could significantly reduce the productions of type I and type III IFN in response to avian Tembusu virus infection in 293T cells [5]. The results of the current study illustrated that the three above mentioned transcriptional factors were significantly upregulated in DEFs at 12 and 24 hpi after DTMUV infection. Meanwhile, these highly expressed transcriptional factors provide favorable conditions for the high production of ILs, IFNs, and chemotactic factors.

## 4. Materials and Methods

### 4.1. Cell Culture and Virus Infection

Duck embryo fibroblasts (DEFs) were obtained from 9-day-old specific pathogen free (SPF) duck embryos (purchased from Harbin Veterinary Research Institute, Harbin, China) according to the manufacturer’s instructions [46], cultured in Dulbecco’s modified Eagle’s medium (DMEM/F-12 1:1) (01-172-1ACS, BI, Kibbutz, Beit Haemek, Israel) at pH 7.2, supplemented with 10% fetal bovine serum (FBS) (04-001-1ACS, BI, Kibbutz, Beit Haemek, Israel), 100 U/mL penicillin and 100 mg/mL streptomycin (P1400, Solarbio Science & Technology Co., Ltd., Beijing, China), and grown in 25 cm^2^ flask (07-8025, BIOLOGIX, Kennebunk, ME, USA) at 37 °C in a 5% CO_2_ cell incubator.

When DEFs reached 80–90% confluency, they were mock-infected or infected with DTMUV (SDSM strain, GenBank Accession No. KC333867.1, which was obtained from our laboratory, the Poultry Disease Lab of Shandong Agricultural University) at a multiplicity of infection (MOI) of 3. After 1.5 h of viral adsorption at 37 °C in a 5% CO_2_ incubator, the inoculum was replaced with maintenance medium (i.e., DMEM/F-12 with 2% of FBS), and then harvested at 12 and 24 hpi, respectively. Three flasks of cells were collected for each treatment at each time point.

Moreover, all animal experiments were performed according to the guidelines of the Committee on Ethics of Animals of Shandong and the appropriate biosecurity, and the Animal Care and Use Committee of Shandong Agricultural University approved the protocol (No. SDAUA-2018-165).

### 4.2. Cell Viability Detection by WST-1 Assay

In this study, in order to evaluate the infection efficiency of DTMUV in DEFs, a WST-1 cell proliferation assay kit (Beyotime, Shanghai, China) was chosen to detect cell viability, as per the manufacturer’s instructions [47]. In total, 2 × 10^3^ DEFs were seeded in in 96-well plates and cultured in DMEM at pH 7.2, supplemented with 2% FBS maintenance medium. Wells containing culture medium alone were used as the control. After incubation at 37 °C in a 5% CO_2_ incubator for 5 h, DEFs were mock-infected or infected with DTMUV at a MOI of 0.01 and maintained for 12 or 24 h. Then, 10 μL of WST-1 solution were added to each well. After 3.5 h of incubation at 37 °C in a 5% CO_2_ incubator, the 96-well plate was examined using a microplate reader (BioTek Instruments, Winooski, VT, USA) at a wavelength of 450 nm. Cell viability was calculated as ((OD_450_ of DTMUV well − OD_450_ of blank control well)/(OD_450_ of PBS well − OD_450_ of blank control well)) × 100%

### 4.3. DEFs Genomic RNA Extraction, Library Construction and Illumina Sequencing

DEFs samples were collected at 12 and 24 hpi (three replicates each) and rapidly stored at −80 °C until further use. Total genomic RNA was extracted using Trizol reagent (Invitrogen, Waltham, MA, USA), as per the manufacturer’s protocols.

The RNA samples were treated with RNase-free DNase I to remove potential genomic DNA, purified using poly-T oligo-attached magnetic beads, and segregated into 200–300-bp fragments. The integrity and concentration of RNA samples were checked using an Agilent 2100 Bioanalyzer (Agilent, Palo Alto, CA, USA), high-quality samples (i.e., A260/A280 ≥ 1.5, A260/A280 ≥ 1.0, and RNA integrity ≥ 7) were used to synthesize first-strand cDNA using reverse transcriptase with 6-base random primers. Second-strand cDNA was synthesized using the first-strand cDNA as a template, and T bases were replaced with U to generate a chain-specific library. Further, library fragments were enriched by PCR amplification, and selection based on fragment size resulted in a 300–400-bp library. Next, 150 bp pair-end readers were performed on the library using an Illumina HiSeq 2000 sequencer at Personal Biotechnology Co., Ltd. (Personal Bio, Shanghai, China).

### 4.4. Transcriptome Data Analysis

Clean data obtained after RNA-Seq were modified by removing adaptors, poly-N sequences, and poor-quality data using Fast-QC software (http://www.bioinformatics.babraham.ac.uk/projects/fastqc/) and Cut adapt software (version 1.2.1, http://code.google.com/p/cutadapt/) [48]. Then, the clean data were assembled *de novo* into unigenes using Trinity software (http://trinityrnaseq.sf.net) [49], and the key parameters (e.g., clean reads, Q20, and Q30) of clean data were also calculated. The assembled unigenes were then mapped to the Peking duck reference genome (duckbase.refseq.v4.fa, http://www.duckbase.org/Download) using TopHat2 software (version 2.1.1, Baltimore, MD, USA) [50,51]. The values of total mapped reads in different samples were also computed by HTSeq software (version 0.9.0, http://htseq.readthedocs.io/en/release_0.10.0/) [52]. Finally, differences in gens expression between mock- and DTMUV-infected groups were calculated and standardized by reads per kilobase of unigene per million mapped reads (FPKM) method [53].

### 4.5. Differential Expression Analysis

Differential expression analysis of data collected in different groups at 12 and 24 hpi was conducted using DESeq software (http://www-huber.embl.de/users/anders/DESeq) [52]. Genes with a *p*-values ≤ 0.05 & |log2(fold change)| ≥ 1 in DESeq analysis were considered as differentially expressed genes (DEGs). To assess the biological function of DEGs, Gene Ontology (GO) enrichment analysis was performed using the Blast2Go software (http://www.blast2go.org/) [54,55]. In this analysis, only categories with a *p*-values < 0.05 were considered significantly enriched in the network. The enrichment results were mainly classified as molecular function, biological process, and cellular component.

The online Kyoto Encyclopedia of Genes and Genomes (KEGG) automatic annotation (http://www.genome.jp/kegg/tool/map_pathway2.html) was used to reveal the location of the DEGs in the different pathways with default parameters [56]. *p*-values < 0.05, denoted statistical significance.

### 4.6. Validation of RNA-Seq Data by qRT-PCR

To verify the reliability of the RNA-Seq data, nine DEGs were randomly selected for quantitative real-time PCR (qRT-PCR) using the specific primers (Table S5). First-strand cDNA was synthesized using total RNA and a PrimeScript™ RT Reagent Kit with gDNA Eraser (TaKaRa, Dalian, China). The PCR assay was conducted using TransStrat Top Green qPCR SuperMix (TransGen Biotech Co., Ltd., Beijing, China) on a 7300 Real-Time PCR instrument (Applied Bio systems, Foster City, CA, USA). Each 20-μL reaction mixture contained 0.5 μL of each primer, 1 μL of DNA template, 10 μL of 2×Trans Strat^®^ Top Green qPCR Super Mix, and 8.0 μL of ddH_2_O. The PCR amplification program followed the following protocol: 94 °C for 30 s, followed by 42 cycles at 94 °C for 5 s, 60 °C for 31 s, and dissociation stage. Each sample had three biological replicates, and the duck glyceraldehyde-3-phosphate dehydrogenase (*GAPDH*) gene served as the endogenous reference gene. The relative expression levels of DEGs were transformed using the 2^−∆∆Ct^ method. All data are shown as the mean ± standard deviation of five replicates. Pearson’s correlation analysis was performed to compare RNA-Seq and qRT-PCR data using the SAS 9.1 software (SAS Institute, Inc., Cary, NC, USA)

### 4.7. Determination of Cytokine Levels in Culture Supernatants Using Enzyme-Linked Immunoassay (ELISA)

In this study, to evaluate the expression of cytokines at the protein level following DTMUV infection, the concentrations of IFN-α, IL-6, IL-8, and IL-12 in culture supernatants were measured using ELISA kits (Mlbio, Shanghai, China), as per the manufacturer’s protocols.

## 5. Conclusions

In summary, our data revealed the transcriptomic profile of DTMUV-infected DEFs for the first time. Upon DTMUV infection, key PRRs (*RIG-I* and *MDA5*) in the immune system were activated, inducing increased production of cytokines (*IFN-α2*, *IL-6*, *IL-8L*, *IL-12B*, *CCR7*, *CCL19*, and *CCL20*), transcription factors or signaling molecules (*IRF7*, *NF-κB*, *STAT1*, *TMEM173/STING* and *TNFAIP3*), and antigen-presenting proteins (*CD44* and *CD70*). These findings broaden our understanding of the immune responses to DTMUV infection, which might contribute to the development of strategies to control the prevalence of DTMUV infection.

## Reference

## Figures and Tables

**Table 1:** Sequencing data generated by an Illumina HiSeqTM 2000 sequencer.

Sample	Raw Reads	Clean Reads	Clean Data (%)	Q20 (%)	Q30 (%)	Total Mapped (%)
12hD1	82,525,576	81,938,158	99.28	97.70	94.29	76.51
12hD2	82,444,204	81,830,302	99.25	97.66	94.24	77.84
12hD3	82,505,912	81,871,548	99.23	97.62	94.15	76.91
12hS1	81,086,676	80,390,534	99.14	97.70	94.28	69.68
12hS2	81,606,422	81,000,120	99.25	97.77	94.41	61.35
12hS3	84,368,222	83,680,924	99.18	97.72	94.30	65.83
24hD1	84,046,382	83,435,382	99.28	97.63	94.15	73.81
24hD2	85,114,912	84,487,666	99.26	97.57	94.03	71.89
24hD3	85,158,520	84,518,936	99.24	97.56	94.03	73.36
24hS1	84,904,016	83,892,832	98.80	97.70	94.24	46.64
24hS2	85,411,010	84,413,208	98.83	97.82	94.46	45.81
24hS3	84,786,930	83,734,226	98.75	97.68	94.17	45.10

## Supplementary Materials

Supplementary materials can be found at http://www.mdpi.com/1422-0067/19/8/2328/s1.
